# CXCR2 Small-Molecule Antagonist Combats Chemoresistance and Enhances Immunotherapy in Triple-Negative Breast Cancer

**DOI:** 10.3389/fphar.2022.862125

**Published:** 2022-04-20

**Authors:** Alaa M. Ghallab, Reda A. Eissa, Hend M. El Tayebi

**Affiliations:** ^1^ The Molecular Pharmacology Research Group, Department of Pharmacology, Toxicology and Clinical Pharmacy, Faculty of Pharmacy and Biotechnology, German University in Cairo, Cairo, Egypt; ^2^ Department of Surgery, Faculty of Medicine, Ain Shams University, Cairo, Egypt

**Keywords:** triple-negative breast cancer, chemoresistance, CXCR2 inhibition, doxorubicin, atezolizumab, AZD5069

## Abstract

Triple-negative breast cancer (TNBC) is the most malignant subtype of breast cancer as the absence of cell surface receptors renders it more difficult to be therapeutically targeted. Chemokine receptor 2 (CXCR2) has been suggested not only to promote therapy resistance and suppress immunotherapy but it also to possess a positive cross-talk with the multifunctional cytokine transforming growth factor beta (TGF-β). Here, we showed that CXCR2 and TGF-β signaling were both upregulated in human TNBC biopsies. CXCR2 inhibition abrogated doxorubicin-mediated TGF-β upregulation in 3D *in vitro* TNBC coculture with PBMCs and eliminated drug resistance in TNBC mammospheres, suggesting a vital role for CXCR2 in TNBC doxorubicin-resistance *via* TGF-β signaling regulation. Moreover, CXCR2 inhibition improved the efficacy of the immunotherapeutic drug “atezolizumab” where the combined inhibition of CXCR2 and PDL1 in TNBC *in vitro* coculture showed an additive effect in cytotoxicity. Altogether, the current study suggests CXCR2 inhibitors as a promising approach to improve TNBC treatment if used in combination with chemotherapy and/or immunotherapy.

## 1 Introduction

Breast cancer (BC) is the most common cancer and one of the leading causes of death in women (Momenimovahed and Salehiniya, 2019). Triple-negative breast cancer (TNBC) is the most lethal subtype of BC characterized by lack of expression of hormonal estrogen (ER+) and progesterone receptors (PR+) and the human epidermal growth factor receptor 2 (HER2+) receptor (Pal et al., 2011). TNBC is thought to be more immunogenic than hormonal subtypes (Rody et al., 2011), and it is consistently associated with poorer survival than non-TNBC (Dent et al., 2007). The major obstacle hindering TNBC effective therapy is the acquired resistance to chemotherapeutics after repetitive and long-term chemotherapeutic administration (Nedeljković and Damjanović, 2019; Gottesman, 2002). Chemoresistance in TNBC is a multifactorial process based on the particular interplay of the tumor microenvironment, drug efflux, cancer stem cells, and bulk tumor cells, where the alterations of multiple signaling pathways govern these interactions (Nedeljković and Damjanović, 2019).

Doxorubicin is one of the anthracycline antibiotics (Barrett-Lee et al., 2009). Presently, it is the most powerful chemotherapeutic agent used to treat breast cancer (Shi et al., 2018). Unfortunately, it has been shown that doxorubicin can cause drug resistance, resulting in poor prognosis and survival in patients since the onset of multidrug resistance in cancer cells usually leads to chemotherapy failure (Li et al., 2005; Lee et al., 2006; Shukla et al., 2010). Studies reported that the interplay between signaling pathways can promote drug resistance through the initiation of proliferation, cell cycle progression, and inhibition of apoptosis (Lee et al., 2006; Abrams et al., 2010). Previous data showed that knockdown of CXCR2 enhances doxorubicin-mediated toxicity in mammary tumor cells. Furthermore, CXCL1, “a CXCR2 ligand,” was found to be overexpressed in doxorubicin-treated mammary tumor cells, which was inhibited following CXCR2 knockdown (Sharma et al., 2013a).

A new weapon against cancer that has elicited durable clinical advances is cancer immunotherapy that inhibits immune-checkpoints; cytotoxic T-lymphocyte–associated protein 4 (CTLA4) or programmed death 1 (PD1) and its ligand, and programmed death 1 ligand (PDL1) has changed the landscape of anticancer treatment (Smyth et al., 2016). Specifically, inhibitors targeting PD1 and PDL1 have shown remarkable clinical efficacy in more than 15 cancer types, including TNBC (Sharma and Allison, 2015; Thomas et al., 2021). This unprecedented success prompted FDA approval of the anti-PDL1 monoclonal antibody (mAb), atezolizumab, for the treatment of TNBC (FDA, 2019). Although, the efficacy of immune checkpoint inhibitors was unusual, many patients did not respond to it. Recent data showed that some patients, who demonstrate encouraging primary responses to immunotherapy, can acquire resistance gradually. Currently, there is an urgent need to predict targets for combination therapy to inhibit or treat resistant tumors (O'Donnell et al., 2017).

Chemokine receptor 2 (CXCR2) is a typical G protein–coupled cell surface chemokine receptor (Wise et al., 2002), which has been found to be highly expressed in various cancers, including breast cancer (Koch et al., 1992). Various studies have observed the role of CXCR2 receptor in tumor aggressiveness, resistance, and immunosuppressive properties (Infanger et al., 2013; Wang et al., 2016; Zhang et al., 2017; Wang et al., 2018). CXCR2 is involved in therapy resistance by maintaining and promoting the migration of cancer stem cells (CSCs), and it is not only suggested as a novel cancer stem-like cell marker for TNBC (Wang et al., 2018) but also known to regulate TGF-β signaling, which is known to promote chemotherapy resistance (Mohammad et al., 2015). Furthermore, there is emerging evidence that the myeloid-derived suppressor cells (MDSCs), recruited to the tumor microenvironment through CXCR2 signaling, play a crucial role in protecting tumors from the cytotoxic T cell–mediated antitumor effect and in suppressing the efficacy of immune checkpoint blockade (ICB) (Talmadge, 2007; Gabrilovich et al., 2012). The blockade of CXCR2 significantly reduced the infiltration of MDSCs and improved the function of cytotoxic T cells in bladder and prostate cancer (Wang et al., 2016; Zhang et al., 2017). In a pancreatic ductal adenocarcinoma model, CXCR2 inhibition was found to augment PD1-inhibition (Steele et al., 2016). CXCR2 inhibition has been proposed as an attractive antitumor treatment not only to enhance immunotherapy but also to intensify the cytotoxicity of chemotherapeutic drugs (Gao et al., 2015).

In an attempt to puzzle out a clinically oriented approach to boost the effect of conventional chemotherapy and immunotherapy in TNBC, we selected a novel, potent, selective, and clinically relevant CXCR2 small-molecule inhibitor “AZD5069.” AZD5069 was developed with the aim of being selective for CXCR2. When measured in a similar system expressing recombinant CXCR1, it was apparent that this drug is around 100-fold more potent against CXCR2 than CXCR1. Furthermore, this drug has recently shown safety and tolerability in patients with chronic obstructive pulmonary disease (COPD) and advanced malignancies (Kirsten et al., 2015; Nicholls et al., 2015). Moreover, AZD5069 has shown rapid absorption in healthy volunteers (Cullberg et al., 2018).

Our study aims at evaluating the impact of targeting CXCR2 in combination with standard chemotherapy and immunotherapy on several outcomes in TNBC using “AZD5069″ as a selective small-molecule antagonist of the human CXCR2.

## 2 Materials and Methods

### 2.1 Patient Sample Collection

In total, 22 pairs of breast cancer tissues and adjacent nonbreast cancer tissues were collected from patients undergoing tumor resection surgery. All tissues are of invasive (Infiltrative) ductal carcinoma (IDC); any other type of breast cancer was excluded. The tissues were kept at −80°C for further use. The blood samples were collected from the same pool of patients where peripheral blood mononuclear cells (PBMCs) were isolated from TNBC patients’ whole blood and were preserved at −80°C for further use. All human materials were obtained with informed consent after the approval of the German University in Cairo and Ain Shams University Ethical Review Committees. The study followed the ethical guidelines of the 1975 Declaration of Helsinki. Patient clinical parameters are presented in Table 1.

**TABLE 1:** Patient characteristics.

Patients (*n* = 22)	Percentage (%)
Sex	
Male (1/22)	5
Female (21/22)	95
Age (years)	
<50 (1/22)	5
>50 (21/22)	95
Subtype	
Invasive ductal carcinoma (IDC) (22/22)	100
Grade	
Grade 2 (15/22)	68
Grade 3 (6/22)	27
Grade 4 (1/22)	5
ER	
ER +ve (14/22)	64
ER −ve (8/22)	36
PR	
PR +ve (13/22)	59
PR −ve (9/22)	41
HER 2/NEU	
HER 2 + ve (6/22)	27
TNBC	
TNBC (6/22)	27
Treatment	
Naïve (12/22)	55
Neoadjuvant chemotherapy (9/22) “alone or combined with herceptin”	41
Targeted therapy “herceptin” (4/22)	18

#### 2.1.1 Blood Collection Procedure

A total of 8 ml blood was drawn from each patient. The blood was stored in EDTA blood collection tubes at 4°C for few hours. Afterward, 6 ml fresh blood was used for isolating PBMCs using the Ficoll separation method. PBMCs of each patient were stored at −80°C for further use. The PBMCs were pooled together during the preparation of the coculture (Section 2.2.1). Plasma was isolated from 2 ml fresh blood by centrifugation and then stored at −80°C for further use.

### 2.2 Cell Culture and Reagents

MDA-MB-231 cells were purchased from Vacsera tissue bank, Egypt. The cells were cultured in DMEM high glucose media supplemented with 10% fetal bovine serum (FBS) and 1% penicillin/streptomycin (pen/strep) at 37°C in 5% CO_2_. After 4 days, when the plate is fully confluent, splitting was performed using 1X trypsin to detach the adherent layer of cells.

#### 2.2.1 *In Vitro* Coculture With PBMCs

The coculture of PBMCs with MDA-MB-231 cells was performed through a series of consecutive steps; PBMCs were isolated from TNBC patients’ whole blood using the Ficoll separation protocol and were preserved in a solution containing 90% FBS and 10% dimethyl sulfoxide (DMSO) at −80°C till further use. Upon coculturing, PBMCs were thawed gently at 37°C in a water bath. The collected PBMCs were then washed by centrifugation, plated at equal density, stimulated by 1% phytohaemagglutinin (PHA), and incubated overnight in a medium consisting of RPMI 1640 supplemented with 10% FBS and 1% penicillin/streptomycin at 37°C and 5% CO_2_. After overnight resting, the PBMCs were treated in triplicate with the doses of AZD5069 (mentioned in Section 2.2.6), and PBMCs were incubated for 48 h at 37°C and 5% CO_2_ after treatment. Finally, PBMCs were cocultured with MDA-MB-231 cells with a ratio 10:1 and were incubated for 72 h.

#### 2.2.2 Mammosphere Formation

MDA-MB-231 were trypsinized and washed in PBS. The cells were counted and visualized on a hemocytometer. If cell clumps were observed, the cells were passed through a 25-gage needle till a single suspension is formed. A total of 8,000 cells per ml were seeded in 24-well ultralow attachment plates (4,000 cell/well) in mammosphere media containing DMEM/F12 supplemented with 2 mM L-glutamine, 100 U/ml penicillin, 100 U/ml streptomycin, 20 ng/ml recombinant EGF, 10 ng/ml recombinant human bFGF, and 1x B27 supplement. The spheres were imaged at Day 7, and spheres greater than 40 µm in diameter were counted as mammospheres and included in the analysis. The number of mammospheres and single cells was counted using trypan blue exclusion. The percent of mammosphere-forming efficiency (M.F.E%) was calculated using the equation: M.F.E (%) = (no. of mammospheres per well)/(no. of cells seeded per well) × 100. All the working wells had approximately the same efficiency as all wells had the same initial seeding density.

#### 2.2.3 Mammosphere *In Vitro* Coculture With PBMCs

TNBC patient PBMCs were treated by the doses of AZD5069 (mentioned in Section 2.2.6). PBMCs were incubated for 48 h at 37°C and 5% CO_2_ after the treatment. The coculturing of PBMCs with MDA-MB-231 mammospheres was performed by adding PBMCs to the mammospheres with the ratio 10:1. The coculture was incubated for 72 h.

#### 2.2.4 RT-PCR Assay

Total RNA was extracted from the tissue of breast cancer patients and their adjacent normal tissue using the BIOzol reagent, according to manufacturer’s instruction. In total, 2 μg of total RNA was used for cDNA synthesis using the cDNA reverse transcription kit (catalog number: 4368813, Thermo Fisher Scientific, United States). Quantification of the CXCR2 gene was carried out using qRT-PCR. For the amplification of the CXCR2 gene, real-time PCR was carried out using the Taqman gene expression assay (catalog number: 4331182, Applied Biosystems, United States) containing the CXCR2 gene, the master mix, and the GAPDH gene expression assay. The geometric mean of the housekeeping gene GAPDH was used as an internal control to normalize the variability in expression levels. The PCR yielded a cycle threshold value (Ct) for each sample. The expression data were normalized to the geometric mean of the housekeeping gene GAPDH to control the variability in expression levels and were analyzed using the 2^−ΔΔCT^ method. The primers used for CXCR2 are as follows:

**Table:** 

Forward	5′-CAGCGACCCAGTCAGGATTTA-3′
Reverse	5′-ACCAGCATCACGAGGGAGTTT-3′

The housekeeping gene GAPDH was used as a control, and primers used are as follows:

**Table:** 

Forward	5′-GTCTCCTCTGACTTCAACAGCG-3′
Reverse	5′-ACCACCCTGTTGCTGTAGCCAA-3′

#### 2.2.5 MTT Viability Assay

For cell viability evaluation in MDA-MB-231 cells and mammosphere post treatment with doxorubicin, triplicate sets of equal densities per plate were seeded in a 24-well cell culture plate under normal growth conditions (37°C and 5% CO_2_). The following day, the cells were treated with step-wise concentrations of doxorubicin (250 nM–2 µM). The MTT experiment was carried out 72-h post treatment using the MTT reagent (catalog number: 11465007001, Sigma Aldrich, Germany). Cell viability was assessed by reading the absorbance at 490 nM using the Victor 1420 multilabel counter plate reader.

#### 2.2.6 Pharmacological Treatment

AZD5069(catalog no: 878385-84-3, MedChem express, United States) was dissolved in DMSO at a concentration of 75 mM, and further dilutions were prepared using DMEM or DMEM/F12. Doxorubicin was dissolved in a free medium of DMEM or DMEM/F12 to prepare an initial concentration of 0.04 mg/ml. Atezolizumab (60 mg/ml vial) was used to prepare a stock solution of (1.2 mg/ml) using free DMEM. For all the parameters measured in the *in vitro* cultures containing PBMCs, 200 nM of atezolizumab (Passariello et al., 2019) was used to treat the coculture, and three different concentrations of AZD5069 (3, 10, or 30 nM) were used to treat the PBMCs before coculturing, where 30 nM is the effective dose for CXCR2 inhibition in PBMCs (Nicholls et al., 2015). For the mammosphere culture, a concentration range of 0–750 nM AZD5069 was used alone or in combination with doxorubicin. In all cultures, doxorubicin (320 nM) was used for treatment, and this dose was concluded from the IC_50_ curve of the drug on MDA-MB-231 cells.

#### 2.2.7 Flow Cytometric Analysis

To evaluate the expression panel of the CXCR2 receptor in the mammospheres, treated and nontreated mammospheres were dissociated and then single-cell suspensions (2.4 × 10^4^ cell/tube) were prepared. For detection of CXCR2, the cells were suspended for 10 min in 0.5% bovine serum albumin (BSA) for blocking; after washing, the cells were kept on a rotator with 2% paraformaldehyde (PFA) for 30 min, washed again, and permeabilized using 0.1% Triton X for 5 min. The cells were washed with PBS and incubated with monoclonal anti-CXCR2 primary antibody (catalog number: sc-7304, Santa Cruz Biotechnology, Germany) (1 µg per one million cells) for 30 min on an ice bucket. After washing with PBS, the cells were incubated with secondary IgG antibody Alexa Fluor 488–conjugated anti-mouse (catalog number: sc-516176, Santa Cruz Biotechnology, Germany) (1 µg per one million cells) for 30 min on an ice bucket. The cells were washed with PBS and then, 10,000 events were acquired for each sample.

#### 2.2.8 ELISA Assay

TGF-β protein was assayed by using the TGF-β ELISA kit (catalog number: MBS266143, My Biosource, United States), according to the manufacturer’s protocol. The absorbance was measured at 450 nM using a Victor 1420 multilabel counter plate reader.

#### 2.2.9 Cytotoxicity Assay

Cytotoxicity of the pharmacological treatments in the *in vitro* culture was detected using the lactate dehydrogenase (LDH) assay kit (catalog number: MBS822351, MyBioSource, United States). The cells were incubated for 72 h after the drug treatment. According to the manufacturer’s protocol, the supernatant of each well was centrifuged, and LDH reagents were used. LDH release was assessed by reading the absorbance at 490 nM using the Victor 1420 multilabel counter plate reader.

#### 2.2.10 Statistical Analysis

Analysis of the *in vitro* experiment was performed using GraphPad Prism 7.02 software. For the purpose of comparison between two populations, Student’s unpaired *t*-test was used, while statistical differences comparing multiple populations were analyzed by analysis of variance (ANOVA). Data were expressed as mean ± standard error of the mean (SEM). A *p*-value less than 0.05 was considered statistically significant. **** = *p* < 0.0001, *** = *p* < 0.001, ** = *p* < 0.01, and * = *p* < 0.05.

## 3 Results

### 3.1 CXCR2 is Highly Expressed in the Tumor Tissue of Breast Cancer Patients

**FIGURE 1:**
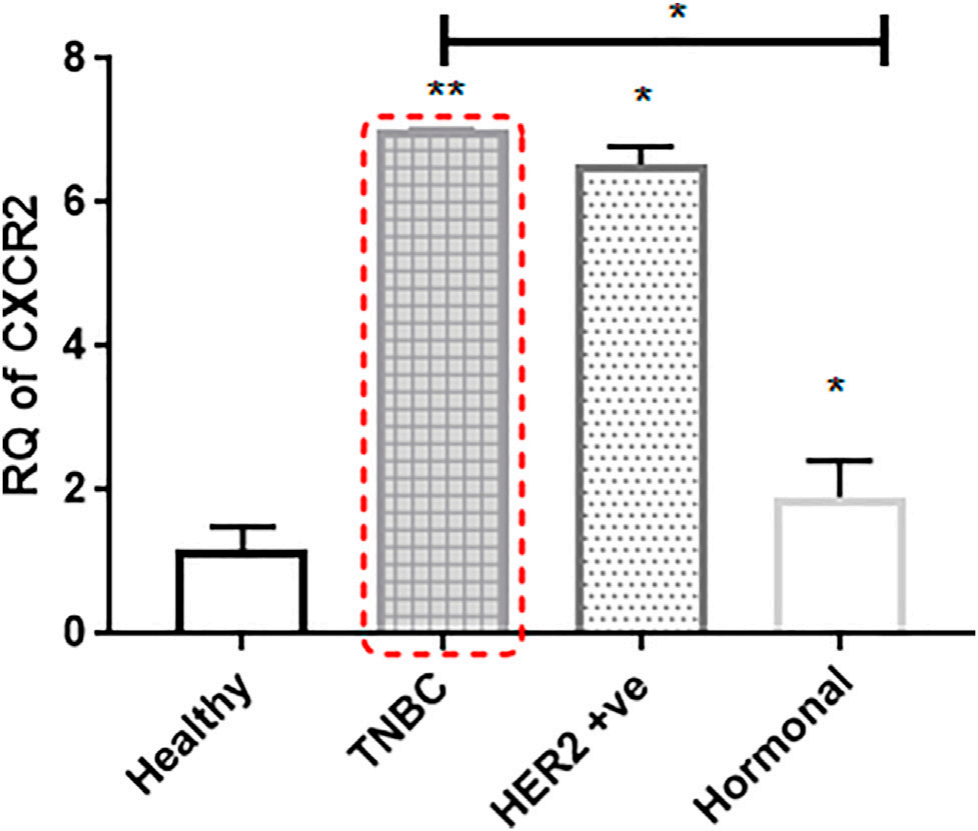
CXCR2 expression is upregulated in the tumor tissue of breast cancer patients. Expression of CXCR2 in different subtypes of BC tissue is compared to the expression in adjacent normal tissue (*n* = 22). RNA was prepared from whole targeted biopsies post resection and from adjacent normal breast tissue to perform RT-PCR experiment. *p* values; unpaired Student’s t-test.

CXCR2 expression is highly upregulated in BC tissues than in adjacent normal tissues. Upon segregation of patient subtypes, TNBC and HER2+ve (luminal B or HER2 enriched) patients showed a dramatic upregulation in CXCR2 expression compared to the controls (*p* = 0.0039 and *p* = 0.0286, respectively). Hormonal patients with ER +ve and/or PR +ve and HER2 −ve receptors (luminal A and normal like subtypes) showed mild significantly upregulated CXCR2 expression (*p* = 0.0179) compared to the controls. CXCR2 expression was significantly higher in TNBC patients than in hormonal patients (*p* = 0.0199) (Figure 1).

### 3.2 Elevation of TGF-β Protein Level in Plasma of Breast Cancer Patients

**FIGURE 2:**
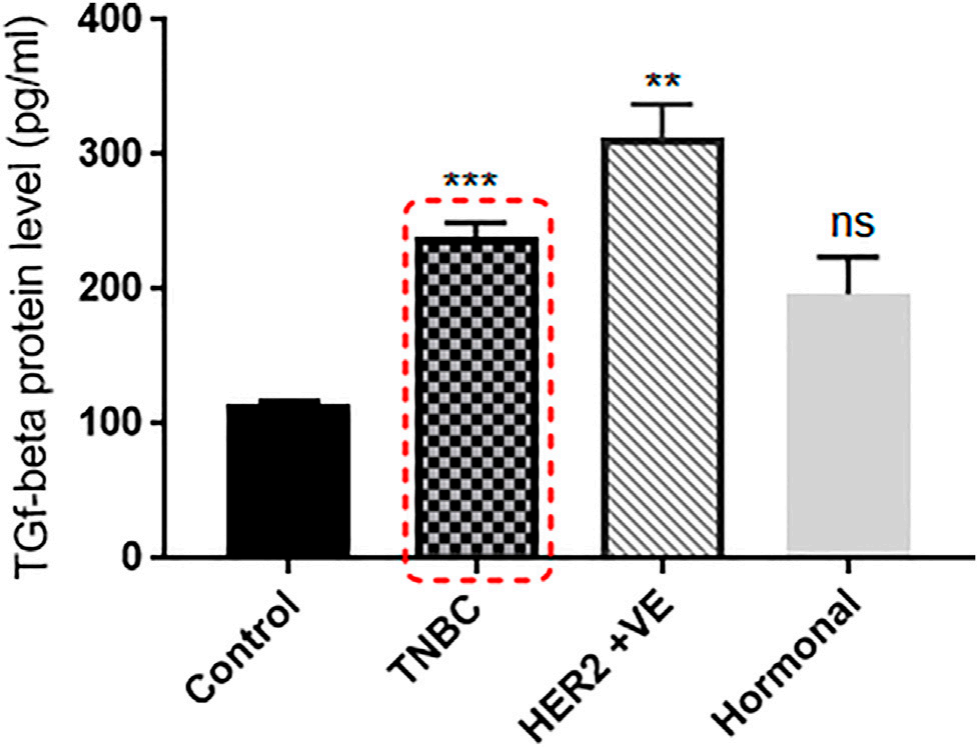
TGF-β protein level is extremely elevated in the plasma of breast cancer patients. TGF-β plasma levels of patients with different BC subtypes (*n* = 22) are compared to the TGF-β levels of healthy individuals. Plasma was separated from whole blood using the Ficoll separation protocol, and TGF-β concentrations were assessed using ELISA. *p* values; unpaired Student’s t-test.

Recent data suggested that TGF-β protein increases with the high expression of CXCR2, and TGF-β is known to possess an impact on drug resistance (Yang et al., 2008). Accordingly, in the same pool of patients, TGF-β protein levels in blood plasma was found to be overexpressed in BC patients than in the healthy control group (all were females, age <55 years, and with no chronic health conditions). TNBC and HER2+ve patients showed significant upregulation in the TGF-β protein level compared to healthy controls (*p* = 0.0004 and *p* = 0.0013, respectively), while hormonal patients with ER +ve and/or PR +ve and HER2 −ve receptors (luminal A and normal like subtypes) showed nonsignificantQ upregulated TGF-β protein levels compared to healthy controls (*p* = 0.0652) (Figure 2).

### 3.3 3D Morphological Characteristics of Mammospheres Generated From MDA-MB-231 Cell Line

A mammosphere formation assay was performed according to Lombardo et al. (2015) to generate primary mammospheres from the MDA-MB-231 cell line. After 7 days, the transformation of the spindle-shaped MDA-MB-231 cells to a larger spheroidal-shaped mammosphere was observed. The images were captured for the MDA-MB-231 cell line before the experiment at ×10 magnification (Supplementary Figure S1), and after the mammosphere formation, the images were captured at ×40 (Supplementary Figures S1B,C) and ×20 magnification. (Supplementary Figures S1D,E).

### 3.4 Comparing the Chemosensitivity Response to Doxorubicin in the MDA-MB-231 Cell Line and Mammospheres

To determine the inhibitory concentration (IC_50_) of doxorubicin in MDA-MB-231 cells, the cell line was treated with step-wise drug concentrations. The cell viability was examined post treatment using the MTT assay and the IC_50_ was calculated. In addition, 320 nM was found to be the drug dose that causes half-maximal inhibitory effect in MDA-MB-231 cells (Supplementary Figure S2). Using the same dose range to treat mammospheres, the MTT assay showed the resistance of the mammospheres to doxorubicin. It was observed that the percent viability increased in mammospheres with higher doses of doxorubicin (Supplementary Figure S2). Upon comparing the cytotoxic effect of 320 nM doxorubicin on both the MDA-MB-231 cell line and the mammospheres, it was observed that although this dose causes 50% inhibitory effect in sensitive cells, it caused a dramatic increase in the cell viability in mammospheres as the viability increased even above the control (*p* = 0.0001) (Supplementary Figure S2). This confirms the molecular changes in the mammospheres and the acquisition of resistance properties.

### 3.5 Doxorubicin Induces Higher CXCR2 Expression Level in MDA-MB-231 Mammospheres

**FIGURE 3:**
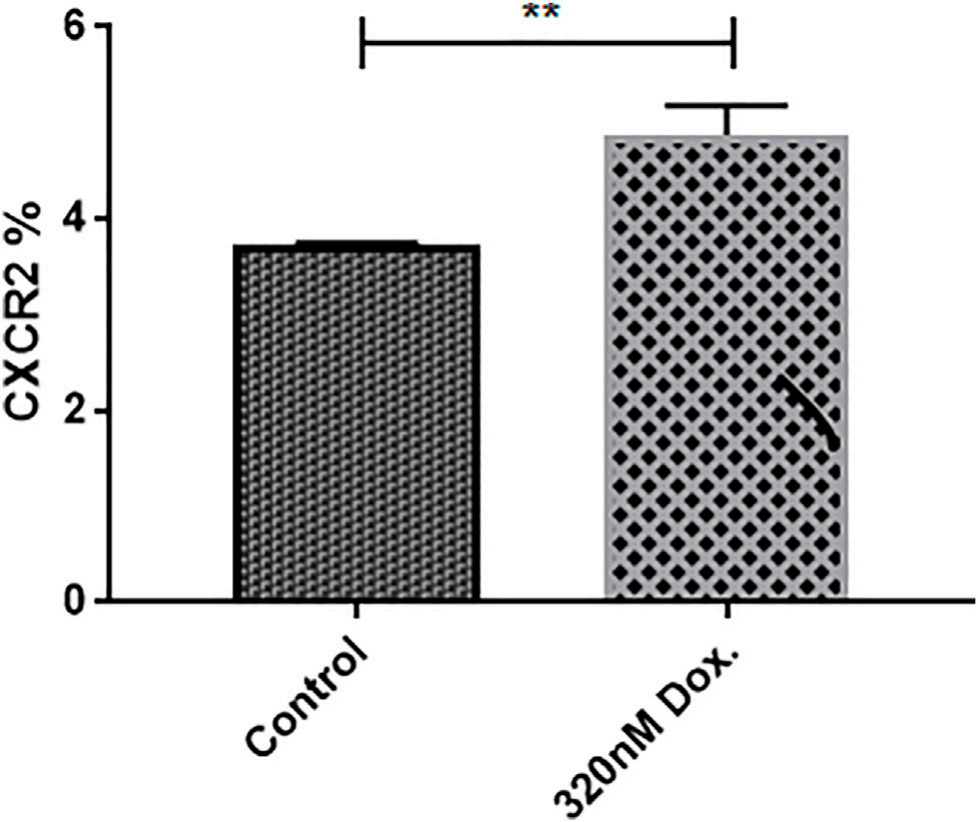
Doxorubicin induces higher CXCR2 expression level in MDA-MB-231 mammospheres. The **c**ytofluorimetric analysis for CXCR2 expression in mammospheres in control and treated conditions; data are expressed as CXCR2 percentage; Dox: doxorubicin. *p* values, unpaired Student’s t-test.

Interestingly, it has been reported that chemotherapy upregulates CXCR2 expression levels in breast cancer cells to increase its aggressiveness (Sharma et al., 2013b). The effect of doxorubicin treatment in the CXCR2 expression in mammospheres was evaluated by cytofluorimetry (Figure 3) and (Supplementary Figure S3). The figure shows a significant increase in CXCR2 expression upon doxorubicin treatment compared to nontreated TNBC mammospheres (*p* = 0.0031). These results could not be confirmed by immunofluorescence due to the very low fraction of CXCR2 positive cells in the bulk population of mammospheres.

### 3.6 AZD5069 Inhibits Doxorubicin-Mediated CXCR2 Overexpression and Restores Primary Levels of the Receptor in TNBC Mammospheres

**FIGURE 4:**
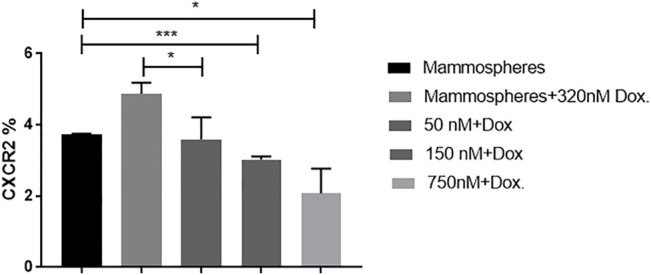
AZD5069 inhibits doxorubicin-mediated CXCR2 overexpression and restores primary levels of the receptor. The cytofluorimetric analysis for CXCR2 expression in mammospheres in control and treated combinations; data are expressed as CXCR2 percentage; Dox: doxorubicin; AZD: AZD5069. *p* values, unpaired Student’s t-test.

To investigate whether inhibiting CXCR2 signaling might affect the CXCR2 upregulation induced by doxorubicin in TNBC mammospheres, mammosheres were treated with doxorubicin (320 nM) alone and combined with several doses of AZD5069. The dose effect of AZD5069 was estimated using flow cytometry. It was found that CXCR2 inhibition by AZD5069 (50 nM) almost completely abrogated the overexpression of the receptor caused by doxorubicin in TNBC mammospheres. The CXCR2 level in mammospheres treated with doxorubicin alone is extremely higher than the CXCR2 level in mammospheres treated with doxorubicin in combination with 50 nM AZD5069 (*p* = 0.0483). In addition, the correlation analysis of AZD5069 doses versus CXCR2 expression showed that higher doses of AZD5069 (150 and 750 nM) decreases the expression of the receptor below primary level (*p* = 0.0008 and 0.0200, respectively) (Figure 4) (Supplementary Figure S4).

### 3.7 Elevated TGF-β Protein Level in the *In Vitro* 3D Culture Post treatment With Doxorubicin

**FIGURE 5:**
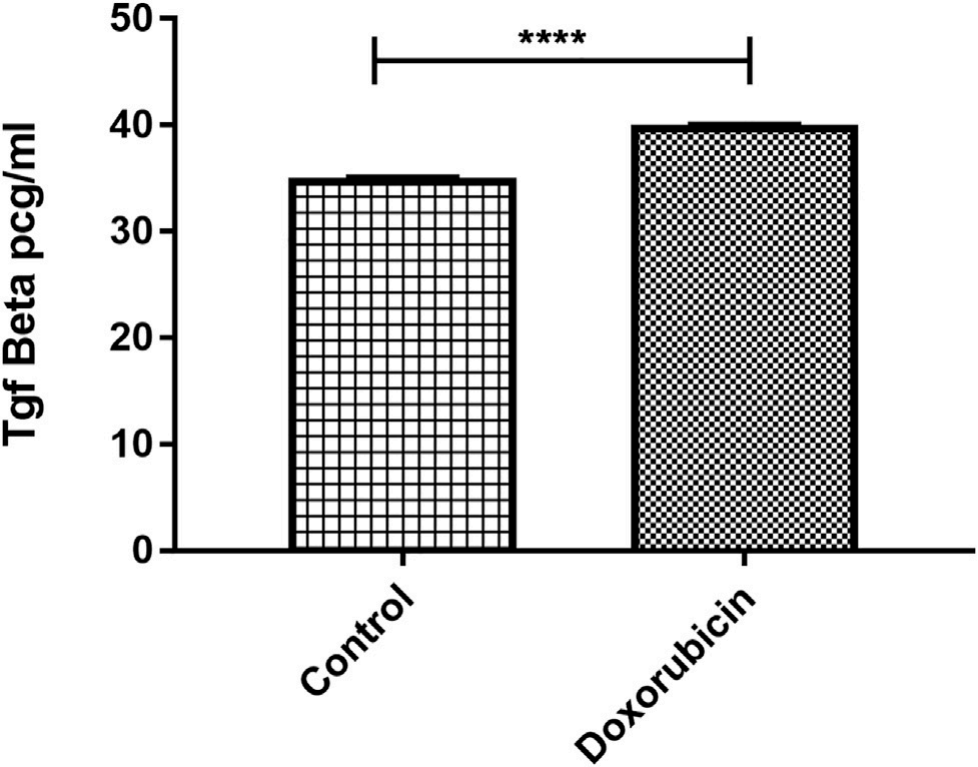
Elevated TGF-β protein level in 3D culture post treatment with doxorubicin. TGF-β levels obtained from ELISA were compared in control and treated conditions. Data are expressed as TGF-β concentration. *p* values; unpaired Student’s t-test.

In an attempt to simulate clinical situation, coculturing of mammospheres with TNBC patient-derived PBMCs was performed in order to explore whether doxorubicin exposure would cause the elevated protein expression of TGF-β. The TGF-β level in the supernatant of the culture treated with 320 nM doxorubicin was estimated using ELISA and was compared to the TGF-β level of the nontreated culture. Statistically significant upregulation in TGF-β was found in the coculture treated with doxorubicin compared to untreated coculture (*p* < 0.0001) (Figure 5).

### 3.8 AZD5069 Prevents Doxorubicin-Mediated TGF-β Upregulation in the MDA-MB-231 Mammospheres *In Vitro* Cultured With PBMCs

**FIGURE 6:**
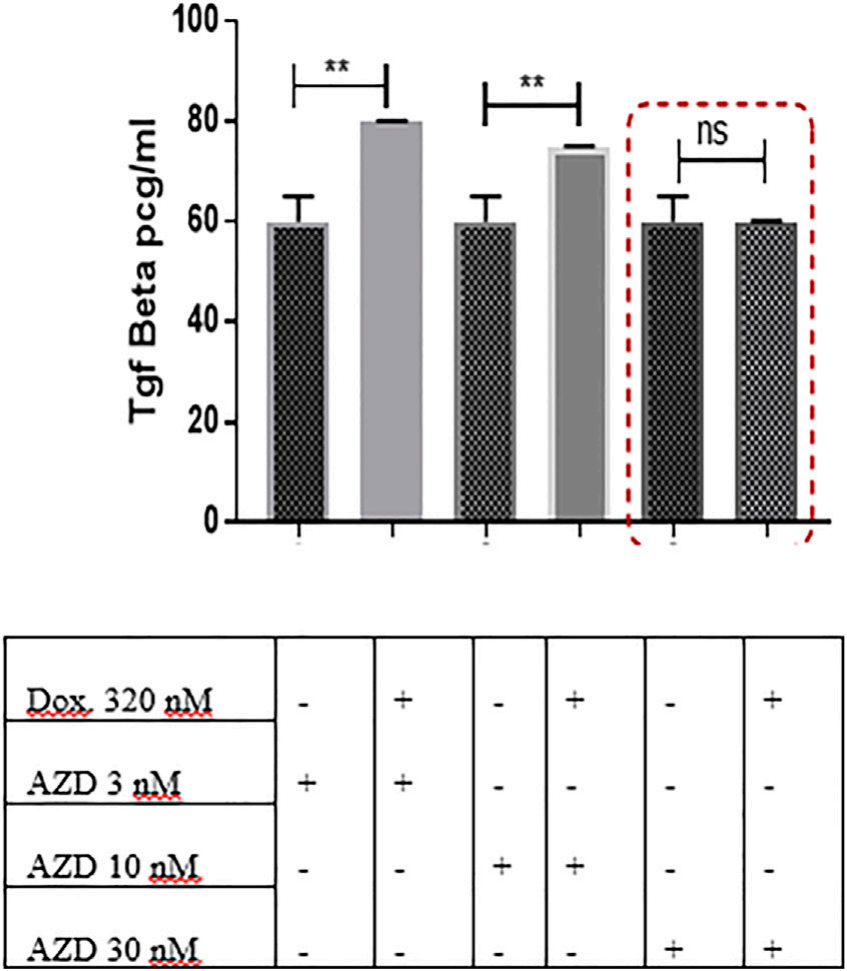
AZD5069 prevents doxorubicin-mediated TGF-β upregulation in 3D culture. TGF-β concentration levels were obtained from ELISA and were compared in different treatment conditions. Data are expressed as TGF-β concentration. Dox: doxorubicin; AZD: AZD5069. *p* values: The TGF-β concentration level of each combined treatment group was compared to the concentration of the corresponding AZD dose alone using unpaired Student’s t-test.

In an attempt to discover whether AZD5069 would have an impact on the TGF-β elevation caused by doxorubicin, TGF-β concentration was measured in the cultures treated with AZD5069 alone and those treated with a combination of AZD5069 and doxorubicin. Doxorubicin induced significant increase in the TGF-β concentration in the cultures treated with small doses of AZD5069, 3 nM AZD5069 (*p* = 0.0023) and 10 nM AZD5069 (*p* = 0.0065), compared to nontreated cultures (Figure 6), while surprisingly, doxorubicin combined with 30 nM AZD5069 did not induce any elevation in the TGF-β concentration, (*p* > 0.9999 = ns) (Figure 6), where 30 nM AZD5069 is the inhibitory dose for CXCL1 ligand-binding inhibition according to the concentration response curve (Nicholls et al., 2015).

### 3.9 CXCR2 Inhibition by AZD5069 Diminishes Doxorubicin Chemoresistance in MDA-MB-231 Mammospheres

**FIGURE 7:**
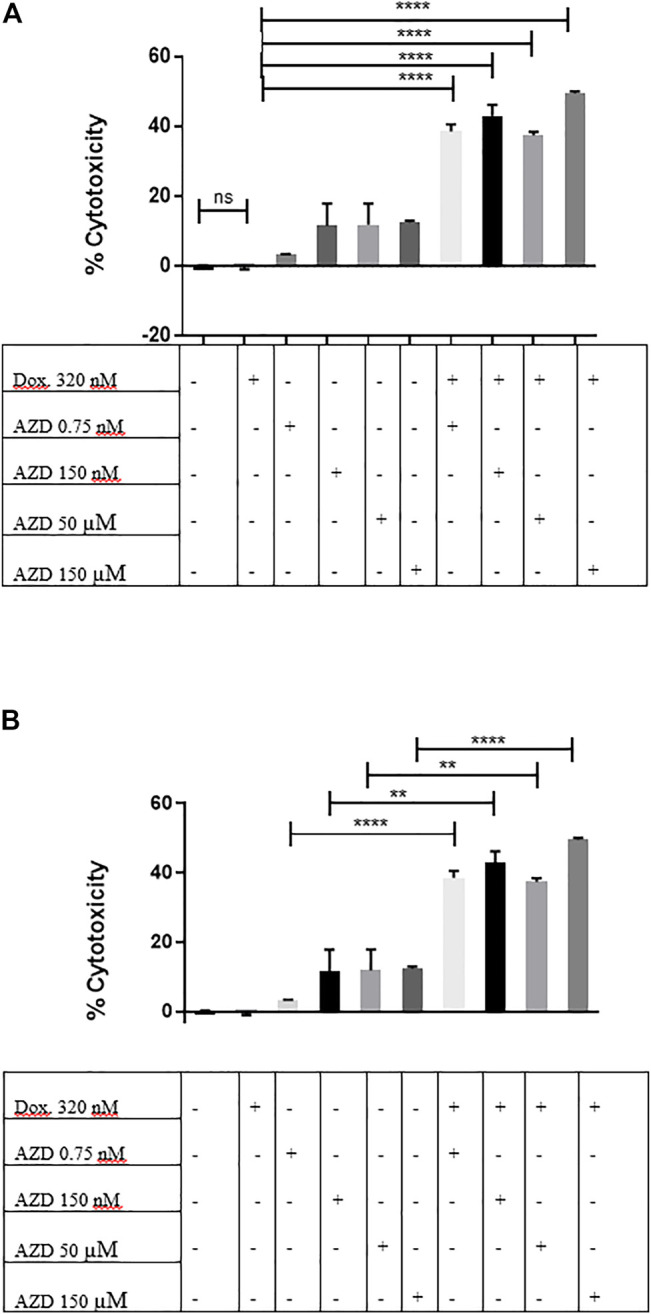
CXCR2 inhibition by AZD5069 diminishes doxorubicin chemoresistance in MDA-MB-231 mammospheres. **(A)** Cell cytotoxicity in control and treated conditions obtained from the LDH assay. Data are expressed as cytotoxicity percentage. Dox: doxorubicin; AZD: AZD5069. *p* values: The cytotoxicity of each combined treatment group was compared to the cytotoxicity of doxorubicin alone using unpaired Student’s t-test. ****p < 0.0001, ns = *p* = 0.2473. **(B)** Cell cytotoxicity in control and treated conditions obtained from the LDH assay. Data are expressed as cytotoxicity percentage. Dox: doxorubicin; AZD: AZD5069. *p* values. The cytotoxicity of each combined treatment group was compared to the cytotoxicity of the corresponding dose of AZD alone using unpaired Student’s t-test. ****p < 0.0001, **p ≤ 0.002.

Recent data suggest the important role of the CXCR2 receptor in maintaining and promoting therapy resistance (Xu et al., 2018a); therefore, would the pharmacological inhibition of CXCR2 signaling inhibit the resistance to doxorubicin in MDA-MB-231 mammospheres? To inhibit CXCR2, AZD5069 was used, the CXCR2 antagonist, at a high, moderate, and low concentrations alone and in association with doxorubicin. While the doxorubicin showed no significant cytotoxic effect on mammospheres (*p* = ns = 0.2473) (Figure 7), the combination of both drugs showed a dramatic significant increase in cytotoxicity than doxorubicin alone (Figure 7) and the corresponding dose of AZD5069 alone (Figure 7B). This clearly showed that the CXCR2 inhibition diminished chemoresistance characteristics in the mammospheres and, thus, enhanced doxorubicin effect.

### 3.10 Targeting CXCR2 Ultimately Fosters Sensitivity to Anti-PDL1 Immunotherapy in MDA-MB-231 Cells Cultured With PBMCs

**FIGURE 8:**
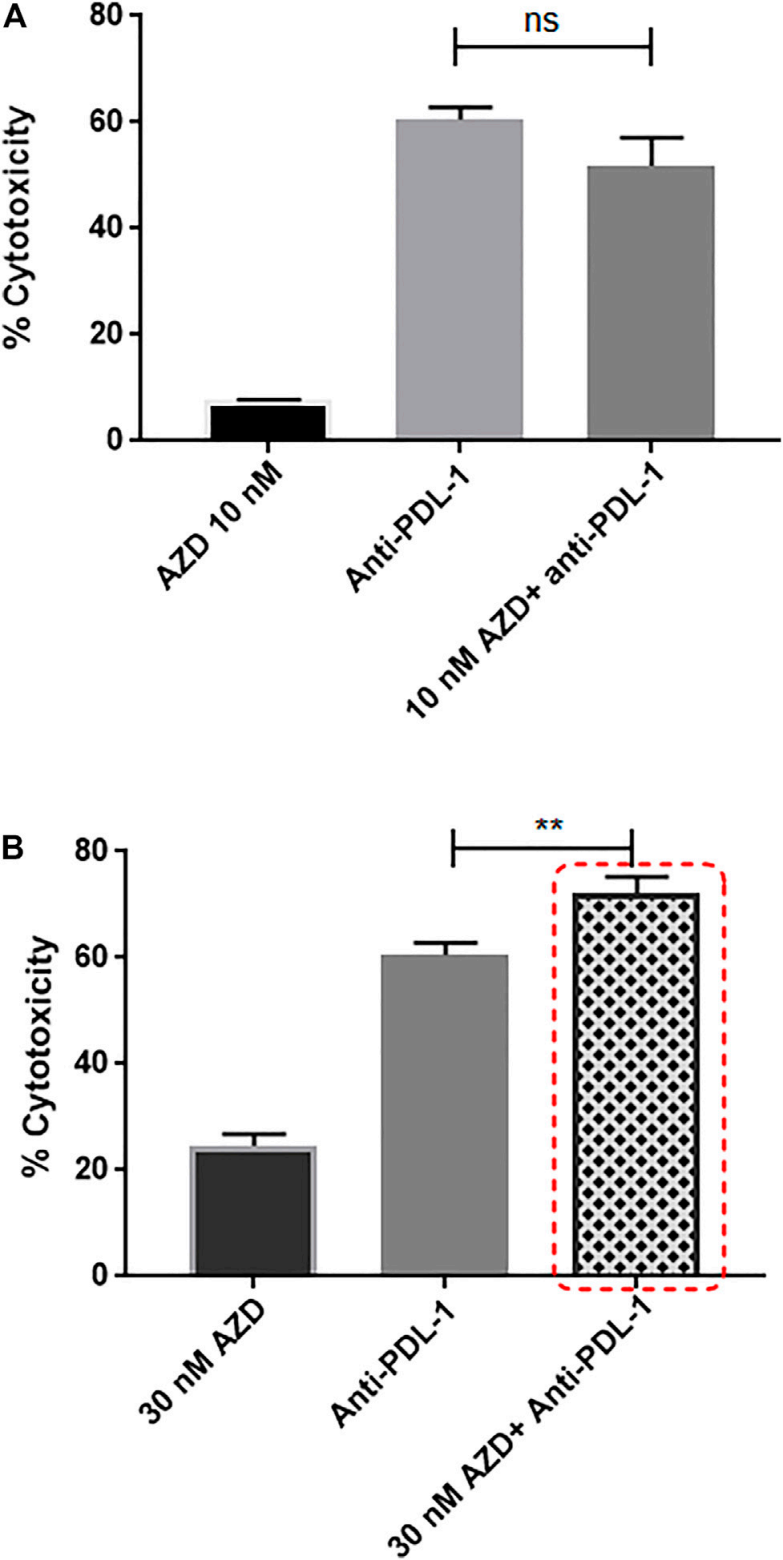
Targeting CXCR2 ultimately fosters sensitivity to anti-PDL1 immunotherapy in MDA-MB-231 cells cultured with PBMCs. **(A)** Cell cytotoxicity in control and treated conditions obtained from the LDH assay. Data are expressed as cytotoxicity percentage. Anti-PDL1: atezolizumab 200 nM; AZD: AZD5069. *p* values. The cytotoxicity of each combined treatment group was compared to the cytotoxicity of atezolizumab alone using unpaired Student’s t-test, ns = *p* = 0.0607. **(B)** Cell cytotoxicity in control and treated conditions obtained from the LDH assay. Data are expressed as cytotoxicity percentage. Anti-PDL1: atezolizumab 200 nM; AZD: AZD5069. *p* values. The cytotoxicity of each combined treatment group was compared to the cytotoxicity of atezolizumab alone using unpaired Student’s t-test, **p ≤ 0.0065.

Recently, anti-PDL1 inhibitors have shown strong potential in multiple tumor types, including TNBC, by overcoming immune suppression and harnessing endogenous antitumor immunity. Knowing that the CXCR2 receptor contributes to promoting immunosuppressive properties, the anti-PDL1 inhibitor combined with CXCR2 small-molecule antagonist was used in a TNBC *in vitro* culture. It was tempting to ask if such a combination would exhibit an additive cytotoxic effect and if CXCR2 inhibitors could enhance sensitivity to anti-PDL1 therapies. In this experiment, TNBC patient–derived PBMCs (treated or nontreated with AZD5069) were cocultured with MDA-MB-231 cells, and the anti-PDL1 inhibitor “atezolizumab” was added to the coculture. Remarkably, the combination of 30 nM AZD5069 (inhibitory dose for the CXCR2 ligand-binding inhibition in PBMCs according to the concentration–response curve) (Nicholls et al., 2015) and 200 nM atezolizumab induced a significant additive effect in cytotoxicity compared to atezolizumab alone (*p* = 0.0065) (Figure 8), while the noneffective dose of AZD5069 (10 nM) (Nicholls et al., 2015) in association with atezolizumab showed no increase in cytotoxicity than atezolizumab alone *p* = ns = 0.0607 (Figure 8).

## 4 Discussion

TNBC is a heterogenous disease which is clinically difficult to manage (Pal et al., 2011) with a clear urge for therapy improvements. CXCR2 converges information from tumor cells and the microenvironment, leading to disease progression, chemoresistance, and immunosuppression, supporting a role for the CXCR2 receptor as a novel therapeutic target. This study aimed at studying the role of CXCR2 inhibitors and their possible use as anticancer drugs for TNBC to diminish chemoresistance and augment immunotherapy. Furthermore, it aimed at demonstrating the impact of CXCR2 inhibition on TGF-β–mediated doxorubicin chemoresistance. In the current study, the effect of AZD5069, a small-molecule CXCR2 antagonist, administered alone or in combination with doxorubicin were assayed *in vitro* on MDA-MB-231 mammospheres. Moreover, AZD5069 was used in combination with atezolizumab, an anti-PDL1 inhibitor, and assayed on the same cell line cultured with PBMCs. Here, we showed that although doxorubicin causes significant elevation in the levels of CXCR2 and TGF-β in 3D culture, CXCR2 inhibition by AZD5069 prevents this elevation and restores their levels back to the nontreated condition. Furthermore, in 3D TNBC culture, we highlighted the benefits of CXCR2 inhibition: not only does the inhibition of CXCR2 diminish doxorubicin chemoresistance but it boosts the efficacy of atezolizumab immune checkpoint inhibitor.

Higher levels of CXCR2 and TGF-β were observed in patients with breast cancer and other types of cancer where they were always correlated with worse clinical outcomes (Ivanović et al., 2006; Li et al., 2011; Gao et al., 2015; Schinke et al., 2015). In our study, the analysis of the CXCR2 expression profile in breast cancer patient tissue biopsies showed that CXCR2 expression is significantly upregulated in patients of the TNBC subtype and other BC subtypes (Figure 1). Furthermore, when we compared the TGF-β protein levels in plasma of breast cancer patients to healthy controls, the TGF-β levels was found to be dramatically upregulated in TNBC patients and Her2+ve patients (Figure 2).

The cellular model used in this study was the triple-negative MDA-MB-231 cells that were not studied in previous experiments using AZD5069. Mammospheres generated from MDA-MB-231 cells were included in this context as they possess chemoresistant properties (Supplementary Figure S1). Although MDA-MB-231 cells were found to express CXCR2 (Supplementary Figure S5), mammospheres were created for the drug-resistant properties they possess. Doxorubicin is a chemotherapeutic agent commonly used in patients with breast cancer and was used in the current study to better understand the effect of CXCR2 inhibition. IC_50_ of doxorubicin was generated from the dose-response curve on MDA-MB-231 cells and was used to treat mammospheres to examine its sensitivity where the IC_50_ of doxorubicin and even higher doses were not able to decrease the viability of the mammospheres (Supplementary Figure S2). In addition, it was observed that doxorubicin promoted mammosphere growth (Supplementary Figure S2B). Though the mechanism was not investigated, studies reported that doxorubicin can induce a higher tumor growth rate in the murine breast tumor model (Christowitz et al., 2019). In the current study, doxorubicin was found to induce higher CXCR2 and TGF-β signaling in the TNBC mammospheres. Although the increase in CXCR2 expression post exposure to doxorubicin is small, it is considered a magnitude of value compared to the normal receptor expression in mammospheres (Figures 3 and 5).

While the use of the CXCR2 inhibitor “AZD5069” in combination with doxorubicin blocked the CXCR2 overexpression and TGF-β elevation mediated by doxorubicin in addition to increasing the chemosensitivity of the mammospheres to doxorubicin (Figures 4, 6 and 7). This is in agreement with other studies linking chemotherapy treatment of TNBC to increased TGF-β signaling (Bhola et al., 2013), where this process was observed to be CXCR2-mediated (Yang et al., 2008). These results are also consistent with previous studies in which Bandyopadhyay et al. (2010); Sharma et al. (2013a) showed an increase in CXCR2 and/or TGF-β levels in aggressive breast cancer cells post treatment with doxorubicin, whereas the targeting of either of them was able to increase the response of cancer cells to doxorubicin. Not only does this elevation occur with doxorubicin but also in epirubicin-resistant TNBC cell lines as reported by a recent study (Xu et al., 2018b).

TGF-β signaling is known to promote TNBC chemotherapy resistance in addition to its immunomodulating effect (Neuzillet et al., 2015); moreover, chemotherapy treatment of TNBC was revealed to increase TGF-β signaling (Bhola et al., 2013). Thus, several TGF-β inhibitors are now being evaluated in clinical trials in breast cancer where they generated either disappointing (NCT01401062) or mixed results (Bogdahn et al., 2011; Giaccone et al., 2015). Several barriers remain ahead of TGF-β–based TNBC therapy, including selectivity and/or specificity issues of TGF-βR inhibitors and the accessibility of TGF-β to monoclonal antibodies (mAbs). Furthermore, the TGF-β pathway suppresses tumorigenesis in early-stage cancers, including breast cancer; therefore, a careful use of inhibitors is needed in order to suppress the tumorigenic arm of the pathway, while fostering the tumor-suppressive one (Shi et al., 2011; Smith et al., 2012). Bierie, B. et al. and Yang, L., et al. observed the positive cross-talk of CXCR2 with the TGF ß pathway. They reported that TGF-βR deletion caused an increase in CXCR2 signaling, which led to increased MDSC recruitment into the tumor microenvironment. These MDSCs caused the production of high levels of TGF-ß (Bierie et al., 2008; Yang et al., 2008). Here, we hypothesized that the inhibition of TGF-β elevation could be achieved through CXCR2 inhibitors. 3D TNBC mammospheres cultured with PBMCs were used to mimic clinical conditions, and data showed that CXCR2 inhibition at optimum doses prevented only doxorubicin-induced TGF-β elevation, thus diminishing TGF-β–mediated chemoresistance without further TGF-β inhibition (Figure 6).

Not only does doxorubicin drive chemoresistance in triple-negative BC cells through CXCR2 and TGF-β upregulation but also induces drug resistance by interacting with other signaling pathways that promote drug resistance, such as MAPK/ERK, PI3K/Akt (Christowitz et al., 2019), and NF-kB, as reported by some studies (Marinello et al., 2019).

It is worth noting that immunotherapy reactivating antitumor immunity has delivered promising results in various tumor types, including TNBC (Sharma and Allison, 2015). Atezolizumab is an anti-PDL1 immune checkpoint inhibitor used in TNBC treatment and was used in the current study to investigate the impact of targeting CXCR2 on the efficacy of anti-PDL1 antibodies. It has been shown in our study that the combination of the CXCR2 antagonist and atezolizumab ameliorates the effect of atezolizumab. Our results suggested that CXCR2 inhibition enhances the effect of immune checkpoint blockade effect in an *in vitro* TNBC model (Figure 8). In concordance with our findings, a recent study showed that CXCR2 inhibition was found to augment programed cell death 1 (PD-1) inhibition in pancreatic cancer (Steele et al., 2016). Altogether, these data are in coherence with previous studies, confirming that the blockade of CXCR2 was able to significantly reduce the infiltration of MDSCs and improves the efficacy of immune checkpoint blockade (Wang et al., 2016; Zhang et al., 2017).

Although the fraction of CXCR2 positive cells in TNBC mammospheres was quite low, this fact must not underscore the importance of CXCR2 signaling in TNBC since their inhibition rendered the TNBC cells more sensitive to doxorubicin and atezolizumab. These data go in line with previous studies reporting the same CXCR2 expression pattern in MDA-MB-231 mammospheres (Brandolini et al., 2015; Wang et al., 2018), where Wang et al. reported that despite the few proportion of CXCR2 positive cells in TNBC mammospheres, these cells were responsible for chemotherapy resistance (Wang et al., 2018).

In conclusion, the present study highlights the role of CXCR2 in inducing chemoresistance and suppressing immunotherapy in TNBC. Our data showed the additive effect of CXCR2 antagonists when combined with conventional chemotherapy or immune checkpoint inhibitors suggesting CXCR2 inhibition as a promising strategy to combat chemoresistance and augment immunotherapy in TNBC. Currently, several small-molecule inhibitors of CXCR2, including “AZD5069”, are being investigated for their anticancer effects in preclinical and clinical studies in several tumor types but not yet investigated in TNBC. More *in vivo* studies are needed to suggest such combination strategies into future clinical trials in dedicated patient population.

## Data Availability

The original contributions presented in the study are included in the article/Supplementary Material, further inquiries can be directed to the corresponding author.
