# Supplementary material for: CXCR2 Small-Molecule Antagonist Combats Chemoresistance and Enhances Immunotherapy in Triple-Negative Breast Cancer
Source: Front Pharmacol. 2022 Apr 20;13:862125. doi: 10.3389/fphar.2022.862125 (PMC9065340; doi:10.3389/fphar.2022.862125)
Supplement: Supplementary file 1 [file DataSheet1.pdf]

Supplementary Data

Supplementary figures:

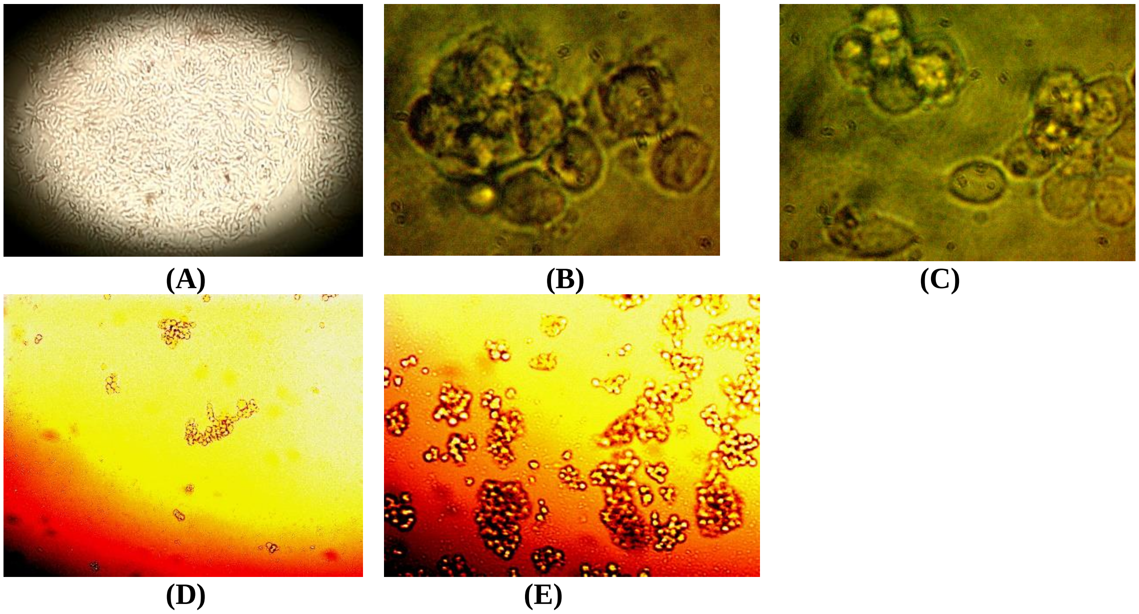

Figure 1 3D Morphological Characteristics of Mammospheres generated from MDA-MB231 Cell line

A) MDA-MB231 cell line, captured by confocal light microscope, lens 10X.

B, C) MDA-MB231 mammospheres, captured by confocal light microscope, lens 40X.

D, E) MDA-MB231 mammospheres captured by confocal light microscope, lens 20X.

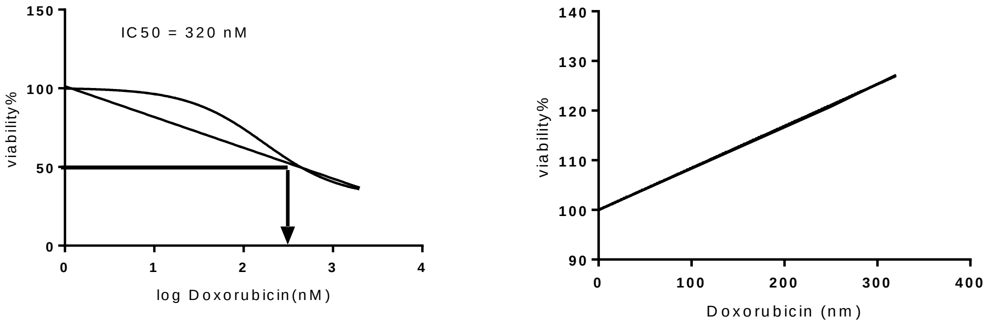

(A) **(B)**

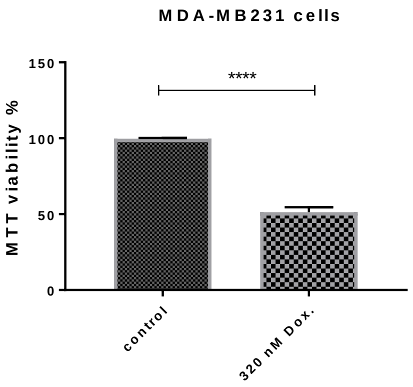

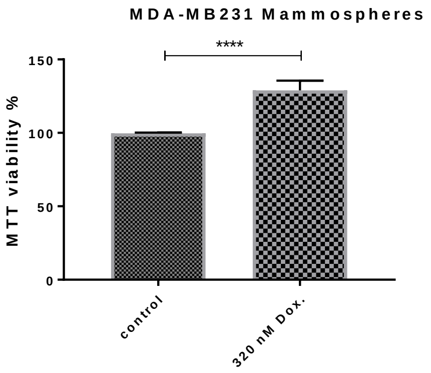

(C) **(D)**

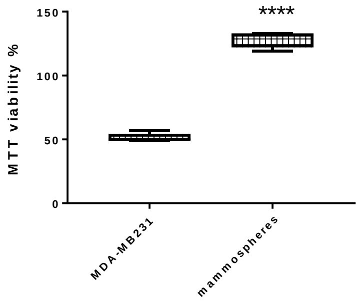

(E)

Figure 2 Comparing the Chemosensitivity Response to Doxorubicin in MDA-MB231 Cell line and Mammospheres.

A) MTT assay for determining the inhibitory concentration (IC50) of Doxorubicin in MDA-MB231 Cells. IC50 was calculated after plotting log of the concentrations on the x-axis while the viability percentage was plotted on the Y-axis, data was fit with nonlinear regression Curve fit.

B) MTT assay for examining the viability in mammospheres post-treatment with step-wise concentrations of Doxorubicin 250-2000 nM (not all doses are shown), data was fit using linear regression Analysis.

C) Compared viability of non-treated and 320 nM Doxorubicin treated MDA-MB231 cells, P values unpaired student’s t-test.

D) Compared viability of non-treated and 320 nM Doxorubicin treated MDA-MB231 mammospheres, P values unpaired student’s t-test.

E) Viability in MDA-MB231 cells post-treatment with 320 nM Doxorubicin is compared to the viability of mammospheres treated with the same dose of the drug P values unpaired student’s t-test.

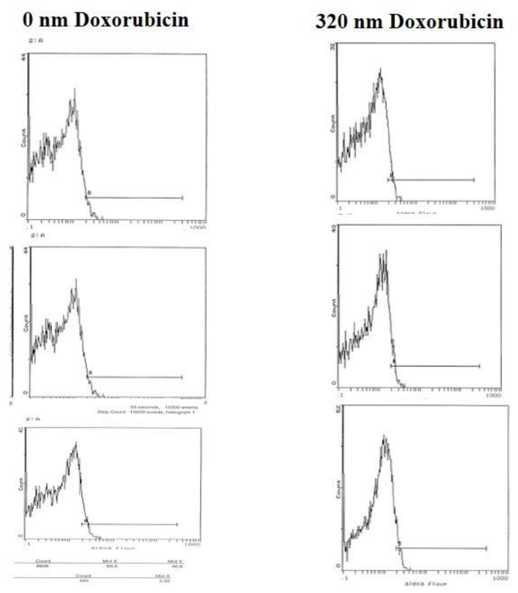

(A)

0 nm Doxorubicin

Hist	Region	ID	%	Count	Mnl X
2	B	B	3.74	363	2.50

(B)

320 nm Doxorubicin

Hist	Region	ID	%	Count	Mnl X
2	B	B	5.23	430	2.40

Figure 3 Doxorubicin Induces Higher CXCR2 Expression level in MDA-MB-231 Mammospheres.(flow cytometric charts)

A) Flow Charts for CXCR2 expression in mammospheres.
B) CXCR2 percentages obtained from flow cytometric analysis.

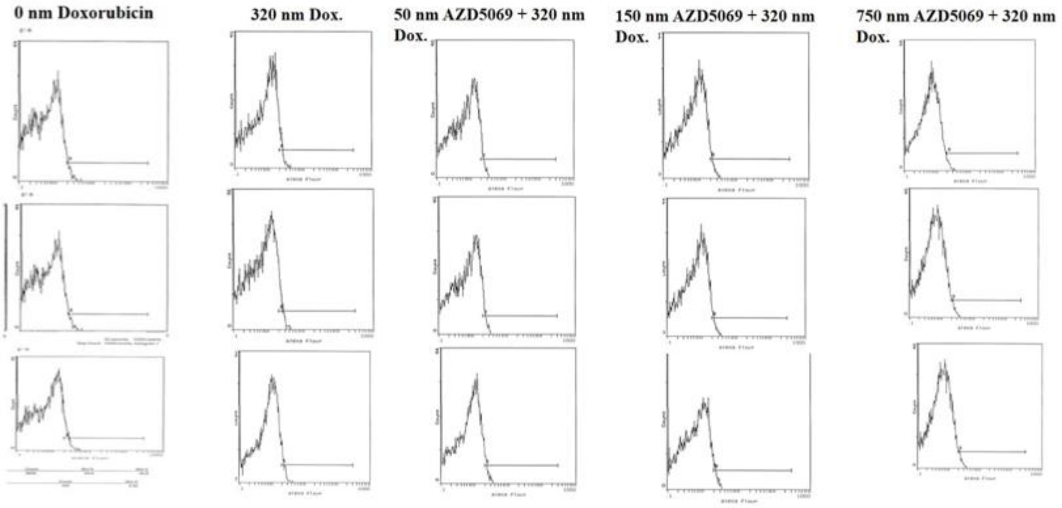

	Hist	Region ID	%	Count	Mnl X
**0 nm Doxorubicin**	2	B B	3.74	363	2.50
**0 nm AZD5069+ 320 nm Dox.**	2	B B	4.73	408	2.39
**50 nm AZD5069+ 320 nm Dox.**	2	B B	3.16	273	2.61
**150 nm AZD5069+ 320 nm Dox.**	2	B B	2.95	258	2.38
**750 nm AZD5069+ 320 nm Dox.**	2	B B	1.59	148	2.46

Figure 4 AZD5069 inhibits Doxorubicin mediated CXCR2 overexpression and restores primary levels of the receptor

(A) Flow Charts for CXCR2 expression in mammospheres.
(B) CXCR2 percentages obtained from flow cytometric analysis.

(A)

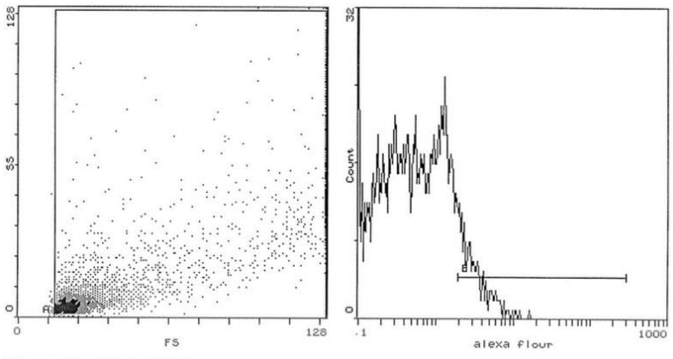

(B)

Hist	Region	ID	%	Count	Mnl X
2	B	B	9.56	755	3.96

Figure 5 CXCR2 Expression level in MDA-MB-231 Cells

A) Flow Charts for CXCR2 expression in MDA-MB-231 Cells.

B) CXCR2 percentages obtained from flow cytometric analysis.
